# Transcription blocking properties and transcription-coupled repair of *N*^2^-alkylguanine adducts as a model for aldehyde-induced DNA damage

**DOI:** 10.1016/j.jbc.2025.108459

**Published:** 2025-03-27

**Authors:** Leen Sarmini, Nataliya Kitsera, Mohammed Meabed, Andriy Khobta

**Affiliations:** 1 Institute of Nutritional Sciences, Friedrich Schiller University Jena, Jena, Germany; 2 Institute of Toxicology, University Medical Center Mainz, Mainz, Germany

**Keywords:** aldehyde-induced DNA damage, alkylation DNA damage, Cockayne syndrome complementation group A (CS-A), Cockayne syndrome complementation group B (CS-B), host cell reactivation (HCR), 1,*N*^2^-ethenoguanine, *N*^2^-ethylguanine, *N*^2^-methylguanine, synthetic DNA adducts, transcription-coupled nucleotide excision repair (TC-NER)

## Abstract

The *N*^2^ position of guanine is a preferential reaction site in DNA for numerous dietary and environmental carcinogens or their electrophilic metabolites, aldehydes arising from lipid peroxidation as well as reactive by-products of normal metabolism. However, DNA repair mechanisms of the resulting covalent adducts in mammalian cells are not well understood, with nucleotide excision repair (NER), base excision repair, and a dioxygenase-mediated damage reversal being discussed as likely pathways. Considering fundamentally different damage recognition principles between the global genome NER and the transcription-coupled (TC)-NER, we here assessed transcription blocking capacities of four synthetic deoxyguanosine (dGuo) adducts of variable size and geometry, using a transfection-based reporter assay. Notably, adducts as different as the aliphatic *N*^2^-ethylguanine, the exocyclic 1,*N*^2^-ethenoguanine, and the bulky polycyclic 3-(deoxyguanosin-*N*^2^-yl)-2-acetylaminofluorene, displayed robust DNA strand–specific transcription-blocking properties. The specific TC-NER components *ERCC8*/*CSA* and *ERCC6*/*CSB* were consistently required for the removal of all transcription-blocking *N*^2^-dGuo adducts, whereas the absence of *XPC* or *DDB2*/*XPE* (both specific to global genome NER) did not compromise the repair capacities in the isogenic human cell models. In contrast, no inhibition of the gene expression was detected for reporter constructs carrying *N*^2^-methylguanine even in the NER-deficient XP-A cell line, suggesting that this adduct is either bypassed with very high efficiency during transcription or repaired by a mechanism different from NER. Collectively, the results identify *N*^2^-dGuo adducts bigger than methylguanine as a structural subclass of transcription-blocking DNA lesions whose repair heavily relies on the TC-NER pathway.

The *N*^2^ nitrogen of deoxyguanosine is an important reaction site for electrophilic metabolites of several classes of occupational, environmental, and dietary carcinogens, including polycyclic aromatic hydrocarbons, aromatic amines, and vinyl chloride. Moreover, it can form adducts by reacting with alkylating agents and aldehydes that arise either in response to stressors or as by-products of physiological metabolism in the cell (1, 2, 3, 4). Dependent on the molecular structure and the number of electrophilic groups in the DNA-damaging species, a vast structural variety of the resulting *N*^2^-deoxyguanosine (*N*^2^-dGuo) adducts has been reported in DNA, as comprehensively reviewed in the literature (1, 2, 4, 5).

Particularly in the case of aldehyde-induced damage, investigation of the repair, toxicity, and tolerance mechanisms is significantly hindered by chemical lability of the relevant DNA adducts in the cellular environment. In contrast, their analogous *N*^2^-alkylguanine adducts are chemically stable. This is exemplified by efficient quantification of acetaldehyde and formaldehyde adducts, *N*^2^-ethylidene-dGuo and *N*^2^-hydroxymethyl-dGuo, in the genome DNA after their reduction with cyanoborohydride to the *N*^2^-ethyl-dGuo and *N*^2^-methyl-dGuo counterparts (6, 7). Importantly, low levels of *N*^2^-ethyl-dGuo were detected in DNA of blood cells from alcoholic patients, suggesting that reduction of *N*^2^-ethylidene-dGuo also occurs under physiological conditions in cells (8, 9). The availability of stable synthetic *N*^2^-alkyl-dGuo compounds offered an opportunity to use them as a proxy for the aldehyde-induced lesions for DNA repair studies. Phosphoramidites of several *N*^2^-alkyl-dGuo adducts have been synthesized and were used to characterize translesion DNA synthesis with purified DNA polymerases (10, 11, 12) or to study their transcriptional bypass (13, 14).

We and others have previously described applications of reporter vectors to characterize the repair of specific types of the incorporated synthetic DNA modifications, based on their transcription-blocking or miscoding properties (15, 16, 17, 18). By the means of carrying the modification in the transcribed strand (TS) the reporter assays are particularly well suited for the detection of transcription-coupled nucleotide excision repair (TC-NER), but are also well applicable to the measurement of global genome-NER (GG-NER), if conducted in a TC-NER–deficient cell line. This allowed identification of several adduct types that strictly rely on TC-NER for repair, including the *N*^*2*^-deoxyguanosine adduct of the experimental carcinogen 2-acetylaminofluorene, and 3,*N*^*4*^-ethenocytosine (εC), induced by a lipid peroxidation product 4-hydroxynonenal (16, 19, 20). Independently, the most recent research has implicated TC-NER in the protection from DNA damage by endogenously arising formaldehyde (21, 22, 23). To explore whether aldehyde monoadducts of *N*^2^-dG may contribute to the observed phenotypes, in this work, we aimed at assessing the outcomes of synthetic MeG and EtG, which represent the reduced forms of adducts generated by formaldehyde and acetaldehyde, in the transcribed DNA. In addition, as a model for the ring-closed subclass of the aldehyde-derived *N*^2^-dG adducts, we have analyzed quantitative contributions of TC-NER and GG-NER to the removal of the exocyclic 1,*N*^2^-ethenoguanine (εG), described previously as a potent block toward human RNA polymerase II (RNAPII) under cell-free conditions (24).

## Results

### Site-specific incorporations of G, MeG, EtG, **ε**G, and AAFG into the noncoding *EGFP* gene region

**Figure 1:**
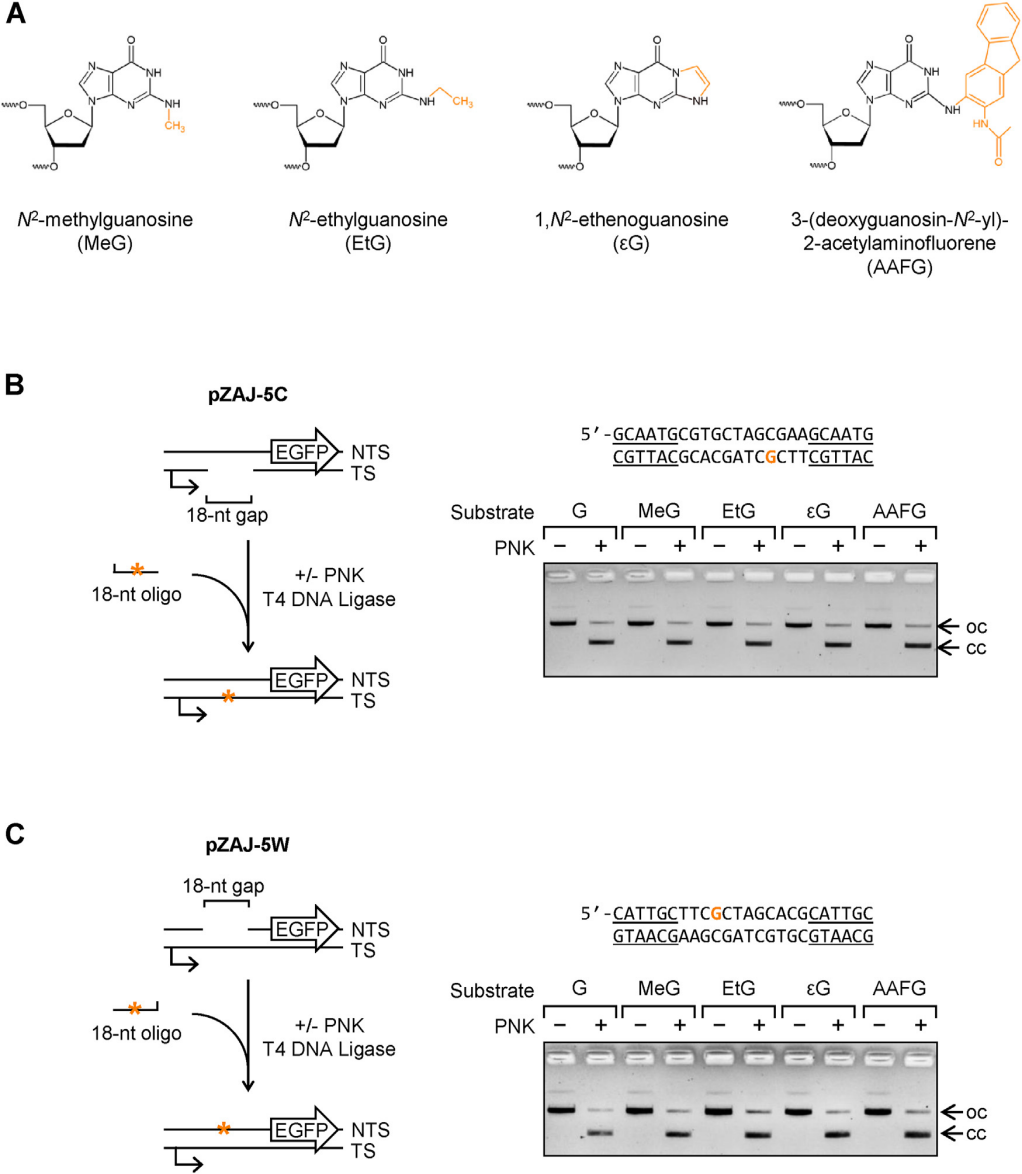
**Site-specific incorporation of *N*^2^-dGuo adducts into the noncoding region of the reporter *EGFP* gene.***A*, chemical structures of four synthetic modifications used for a sequence-specific insertion into reporter vectors. Groups added at *N*^2^ are evidenced with *amber color*. *B*, incorporation of synthetic 18-nt oligonucleotides accommodating the specified modification types (*amber asterisk* in the scheme) into the transcribed *EGFP* strand (TS). The modified nucleotide (*amber “G”*) and surrounding DNA sequence (with BsrDI recognition sites *underlined*) are shown above the agarose gel image. A reference construct for a fully efficient transcriptional bypass in the reporter assay was generated by the incorporation of a modification-free synthetic strand, containing 2′-deoxyribo-guanine (G) at the specified position. A representative agarose gel shows seamless ligation of deoxyribo-oligonucleotides with the specified modifications into pregapped pZAJ-5C vector. Their efficient incorporation was confirmed by inhibition of conversion of the open circular plasmid (oc) into the covalently closed form (cc) when polynucleotide kinase (PNK) was omitted from the ligation reactions (25, 26). *C*, efficient incorporation of the same set of synthetic oligonucleotides as in “B” into the pZAJ-5W vector with pregapped nontranscribed *EGFP* strand (NTS). dGuo, deoxyguanosine.

To characterize the effect of the size of the substituting group on the property of *N*^2^-dGuo adducts to interact with elongating RNAPII in human cells, we chose a reporter-based approach. Chemical structures of the listed adducts are shown in Figure 1*A*. We incorporated synthetic oligonucleotides containing guanine (G), *N*^2^-methylguanine (MeG), *N*^2^-ethylguanine (EtG), or 1,*N*^2^-εG into the TS of a plasmid-borne *EGFP* gene expressed under the control of the cytomegalovirus immediate early promoter (Figs. 1*B*, S1). In addition to the investigated adducts, we have generated *EGFP* constructs containing synthetic 3-(deoxyguanosin-*N*^2^-yl)-2-acetylaminofluorene (AAFG), which was previously classified as a potent transcription-blocking lesion (16). Importantly, the modifications were placed in the untranslated region of the transcribed portion of the gene. Such a design assures that ribonucleotide misincorporation or skipping by the elongating transcription complexes at the damage site would not affect the protein structure. Thereby, any changes of the EGFP expression should directly reflect the transcription rate of the gene. In addition, to distinguish between the RNAPII-mediated and -independent modes of action, we included control reporter constructs carrying the analyzed modifications in the nontranscribed strand (NTS) (Figs. 1*C*, S1).

Analyses of the generated constructs by gel electrophoresis showed that all synthetic strands were efficiently incorporated into the reporter vectors (Fig. 1, *B* and *C*). As there are no enzymes, which would specifically detect the presence of MeG, EtG, εG, or AAFG in plasmid DNA, we instead validated the incorporation of the respective oligonucleotides by inhibition of self-ligation of vector DNA (25, 26). Indeed, no covalently closed circular DNA was formed in the ligation reactions when synthetic DNA strands (which lack the 5′-phosphate) were incubated in the absence of a polynucleotide kinase. Taking into account the band detection sensitivity limit, the result indicated that all adducts were incorporated into plasmid DNA with efficiencies of more than 97%. Phosphorylation with polynucleotide kinase increased the rates of formation of the covalently closed form to at least 90%, indicating that all modification-containing oligonucleotides were correctly accommodated within the gap, with both junction sites sealed.

### EtG and **ε**G abrogate the EGFP gene expression in NER-deficient cells in a strand-specific manner

**Figure 2:**
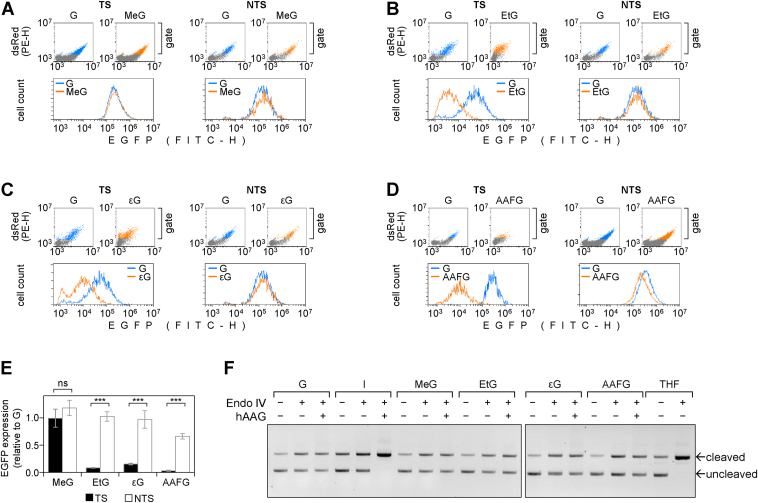
**Assessment of transcription inhibitory capacities of four different *N*^2^-dGuo adducts in the XP-A cell line GM04312.***A–D*, representative flow cytometry data of the EGFP expression in the presence of the indicated adducts in either DNA strand (TS or NTS): (*A*) MeG; (*B*) EtG; (*C*) εG; (*D*) AAFG. The obtained EGFP/DsRed scatter plots are shown pairwise for the specified adducts (MeG, EtG, εG, or AAFG) and the modification-free controls (G). Transfected cells were gated based on the DsRed expression (as highlighted with *blue and amber colors*) to derive the EGFP fluorescence distribution plots, shown, overlaid, for each pair of constructs under the respective scatter plots (*E*) Quantification of the EGFP expression in cells transfected with constructs carrying the specified adducts in the TS compared to the NTS. Median EGFP fluorescence values were normalized relative to “G” controls and plotted as mean ± SD of *n* = 6 (TS) or *n* = 3 (NTS) independent experiments. Two-tailed homoscedastic Student’s *t* test: not significant (ns) or *p* < 0.001 (∗∗∗). *F*, assessment of hAAG activity toward the specified *N*^2^-dGuo adducts in comparison to 2′-deoxyinosine (I). Plasmid constructs containing single synthetic modifications were incubated with hAAG in the presence of endonuclease IV (Endo IV), and strand cleavage was assessed by electrophoresis in the presence of ethidium bromide. hAAG was added in excess, as demonstrated by saturating strand cleavage at I, whereas the excess of Endo IV was confirmed by complete cleavage or an apurinic/apyrimidinic site analog tetrahydrofuran (THF). The gel image is rearranged for clarity of presentation: because of capacity limits, samples were loaded in two rows. εG, ethenoguanine; AAFG, 3-(deoxyguanosin-*N*^2^-yl)-2-acetylaminofluorene; EtG, ethylguanine; dGuo, deoxyguanosine; hAAG, human AAG; MeG, methylguanine; NTS, nontranscribed strand; TS, transcribed strand.

Transcription blocking capacities of DNA modifications can be measured in a cellular context based on the expression levels of a reporter gene carrying the modification in question in the TS (16). However, in repair-proficient host cells, the effects of DNA modifications on transcription may be masked or quantitatively distorted by concomitant damage removal from the transcribed template (20). Considering that there is only fragmented knowledge about repair mechanisms of *N*^2^-alkyl G adducts in mammalian cells, we performed the reporter expression analyses in a NER-deficient XP-A (GM04312) cell line (27). Of all analyzed *N*^2^-dGuo adducts, only MeG did not significantly affect the EGFP expression (*p* = 0.9, two-tailed heteroscedastic Student’s *t* test), indicating that this modification either does not interfere with the transcription processes or is repaired with a very high efficiency in the host cell line (Fig. 2*A*). In contrast, EtG resulted in a very strong (13-fold, *p* = 1.6 × 10^−12^) decrease of the EGFP expression, indicating a potent inhibition of transcription by this DNA modification (Fig. 2*B*). With a 7-fold reduction of the EGFP signal (*p* = 2.4 × 10^−10^) the effect of εG was also highly significant, thereby indicating a clear negative effect on the gene expression (Fig. 2*C*). Finally, and as expected (16), the previously characterized transcription-blocking NER substrate AAFG abolished the EGFP expression almost entirely, as deduced from a 33-fold decrease (*p* = 7.9 × 10^−28^) of the specific fluorescence signal in the transfected cells (Fig. 2*D*).

To assess whether inhibition of the *EGFP* expression is attributable to interaction of the investigated *N*^2^-dGuo modifications with elongating transcription complexes, we performed parallel transfections to measure the effects of the same modifications incorporated into the NTS (Fig. 2, *A*–*D*). MeG did not cause a significant change in the EGFP expression (*p* = 0.16), in agreement with the absence of an effect of this DNA modification in the TS. In contrast, very significant differences were observed between TS and NTS for the remaining three adduct types, clearly indicating that their effects on the EGFP expression level are mediated by the impairment of gene transcription and not by a diminished delivery or an aberrant processing of adducted DNA in an extranuclear compartment (Fig. 2*E*). Importantly, if present in the NTS, EtG (*p* = 0.68), and εG (*p* = 0.77) no longer caused any significant effect on the EGFP signal (relative to unmodified dGuo), indicating a strictly TS-specific mode of action of these adducts.

Of all *N*^*2*^-dGuo modifications analyzed, only AAFG caused a mild decrease of the EGFP signal when present in the NTS (Fig. 2, *D* and *E*). Thus, while EtG and εG apparently act *via* blockage of the elongating transcription complexes only, the effects of AAFG seem to be more complex. A discernable effect of AAFG in the NTS on the gene expression (a 1.5-fold decrease) suggests that, besides RNAPII blockage, some additional mechanism may contribute to the declined EGFP expression. On the other hand, AAFG is far more potent when placed in the TS (causing a 33-fold decrease of the EGFP signal); strongly suggesting that transcription blockage is the main cause of the impaired gene expression in the case of this DNA modification as well.

### MPG does not significantly contribute to the repair of transcription-blocking *N*^2^-dGuo adducts

Based on the biochemical evidence, it has been inferred that repair of 1,*N*^2^-εG can take place by base excision repair (BER) mediated by the monofunctional *N*-methylpurine DNA glycosylase (MPG), also known as AAG or ANPG (28). However, pronounced differences between the TS and NTS in the effects imposed by εG, EtG, or AAFG on the *EGFP* gene expression (Fig. 2*E*), seem to be hard to reconcile with any significant contribution of BER to repair of these adducts in the cellular context. To re-evaluate the capacity of human AAG (hAAG) to excise the *N*^2^-dGuo modifications in a biochemical assay, we incubated plasmid constructs containing MeG, EtG, εG, AAFG, or a *bona fide* AAG substrate 2′-deoxyinosine with hAAG (Fig. 2*F*). Since hAAG is a monofunctional DNA *N*-glycosylase, the resulting apurinic lesion was cleaved by coincubation with endonuclease IV under conditions preliminarily determined based on exhausting excision of its substrate tetrahydrofuran. Even though incubation with excess of hAAG resulted in a complete cleavage of the plasmid substrate containing single 2′-deoxyinosine, no appreciable increase in the fraction of nicked plasmid DNA was detected in substrates carrying either of the *N*^2^-dG adducts. The results indicate that AAG activity toward the analyzed *N*^2^-alkylG adducts is absent or negligible, which corroborates the conclusion made previously, based on the *EGFP* gene expression inhibition.

### Repair of transcription blocking *N*^2^-dGuo adducts is *XPA*-dependent

**Figure 3:**
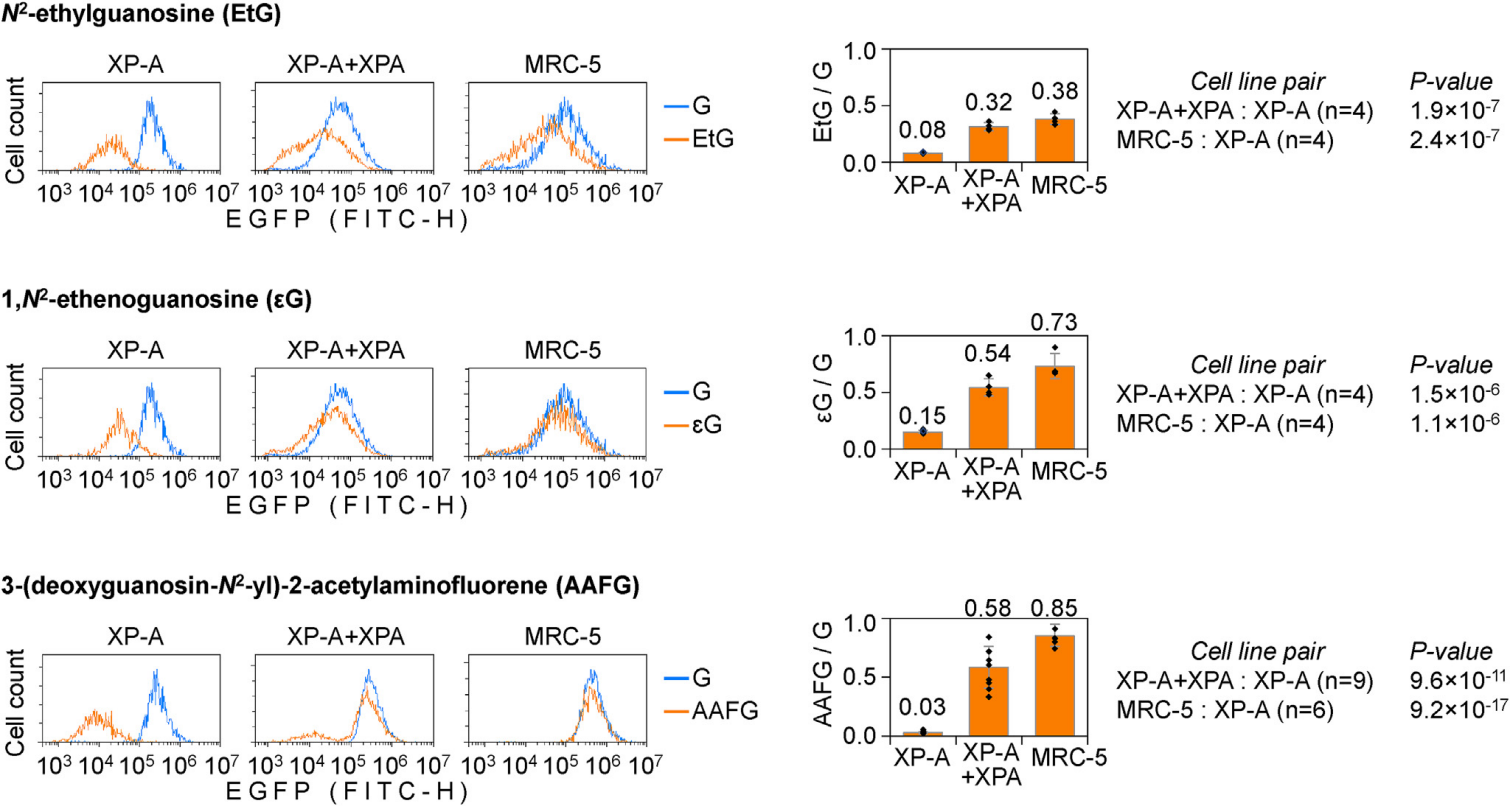
**Host-cell reactivation of reporter constructs carrying the specified *N*^2^-dGuo adducts in the TS in cell lines with different NER statuses: XP-A, the isogenic XPA-complemented (XP-A + XPA), and MRC-5.** Representative fluorescence distribution plots show EGFP expression for the specified *N*^2^-dGuo adducts (EtG, εG, AAFG) overlaid with the adduct-free controls (G). *Bar charts* on the *right* show quantification of the EGFP expression, relative to G (mean of *n* ≥ 4 independent experiments with all cell lines transfected in parallel, ±SD, *p* values calculated by Student’s two-tailed *t* test). AAFG, 3-(deoxyguanosin-*N*^2^-yl)-2-acetylaminofluorene; dGuo, deoxyguanosine; εG, ethenoguanine; EtG, ethylguanine; NER, nucleotide excision repair; TS, transcribed strand.

To assess whether EtG and εG adducts can be removed by NER, we assessed reactivation of the EGFP expression in a NER-proficient MRC-5 cell line and in an XP-A–derived cell line complemented with a functional *XPA* gene. Expression rates of constructs containing EtG or εG in the TS were significantly improved in both MRC-5 and the XPA-complemented cells compared to the XP-A cell line (Fig. 3). In the XPA-complemented cells, the reactivation rates were overall somewhat lower than in MRC-5, which can be attributed to the presence of a minor subpopulation of NER-deficient cells, identifiable by absent repair of the reference NER substrate AAFG (second peak distinguishable in the fluorescence distribution plots). Nonetheless, the improvement of the host cell reactivation (HCR) rates by the *XPA* transgene, highly significant for both EtG and εG, indicated that, like AAFG, they are repaired in an XPA-dependent manner.

Interestingly, even in fully NER-proficient MRC-5 cells, reactivation rates of EtG (38.26% ± 4.75%) were noticeably smaller than for AAFG (85.34% ± 9.3%) or εG (73.19% ± 11.09%) (Fig. 3). Also in XPA-complemented cells, reactivation of the EtG construct was relatively weak (31.66% ± 3.57%) in comparison to εG (54.36% ± 7.67%) or AAFG (58.36% ± 1.77%). This may indicate that, despite the equivalent transcription blocking capacities, repair of EtG in cells is less efficient than NER of bulkier *N*^2^ modifications, such as εG and AAFG.

### TC-NER is essential for the removal of EtG and **ε**G

**Figure 4:**
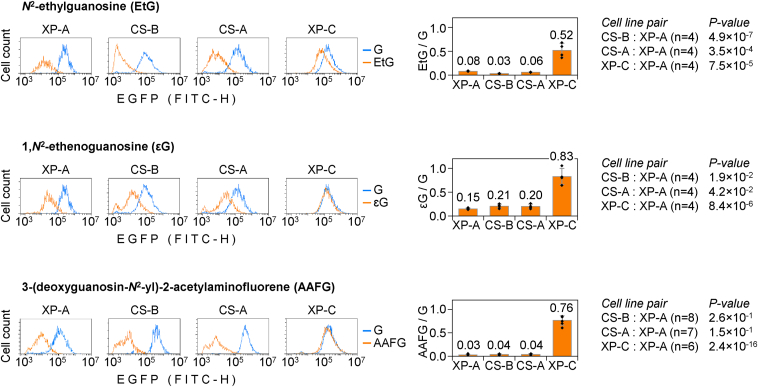
**Host-cell reactivation of reporter constructs carrying the specified transcription-blocking *N*^2^-dGuo adducts in cell lines derived from patients of the specific TC-NER (CS-B, CS-A) or GG-NER (XP-C) complementation groups.** XP-A cells, deficient in both NER subpathways, were transfected for comparison with the same constructs. Representative fluorescence distribution plots and quantification of the EGFP expression relative to the adduct-free construct (mean of *n* ≥ 4 independent experiments, ±SD). CS, Cockayne syndrome; dGuo, deoxyguanosine; TC-NER, transcription-coupled nucleotide excision repair.

Considering that both TC-NER and GG-NER activities can be detected by the reporter gene reactivation assay (16, 20), we further questioned, what are specific contributions of the individual NER subpathways to the repair of EtG and εG. To this end, we compared the reporter reactivation capacities of cell lines derived from patients of different NER complementation groups (TC-NER–deficient Cockayne syndrome B [CS-B] and Cockayne syndrome A [CS-A] in contrast to a GG-NER–deficient XP-C), using the obligate TC-NER substrate AAFG as a positive control. In both TC-NER–deficient cell lines, single EtG in the TS led to an essentially complete (as in XP-A cells) loss of the EGFP signal (Fig. 4). In contrast, a robust recovery of the EGFP expression was observed when the EtG reporter construct was transfected to GG-NER–deficient cells (from 8.05% ± 0.54% in XP-A to 51.56% ± 14.81% in XP-C, both values relative to expression levels of the adduct-free construct). Similarly, for εG in the same position, there was almost no difference between the XP-A and CS (CS-B and CS-A) cell lines, while the XP-C cell line, again, showed a clear recovery of the EGFP expression (to 82.98% ± 1.70% against 15.17% ± 1.37% in XP-A), indicating an efficient TC-NER and implying that GG-NER did not significantly contribute to overall repair.

**Figure 5:**
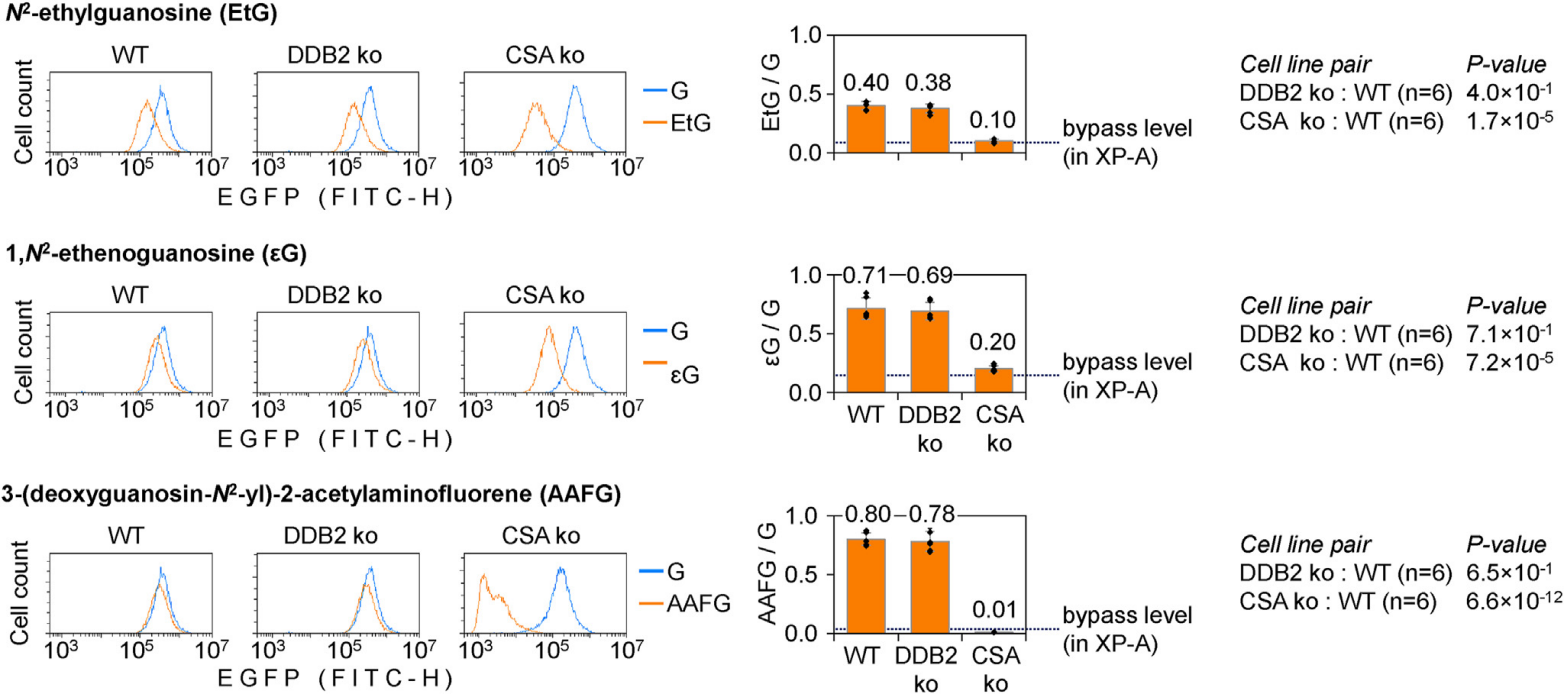
**Effects of *DDB2* and *CSA* gene knockouts in HeLa cells on the host-cell reactivation of reporter constructs carrying the specified *N*^2^-dGuo adducts in the TS.** The knockout cell lines are labeled as “DDB2 ko” and “CSA ko,” and the parental HeLa cell line as “WT.” Representative fluorescence distribution plots and quantification of the EGFP expression relative to the adduct-free construct (mean of *n* = 6 independent experiments, ±SD). *Dotted line* shows the lower detection boundary for the repair activity, extrapolated from the NER-deficient XP-A cell line. CSA, Cockayne syndrome A; EtG, ethylguanine; dGuo, deoxyguanosine; NER, nucleotide excision repair; TS, transcribed strand.

The inability to reactivate reporter constructs containing synthetic EtG and εG, manifested by two independent CS complementation groups, strongly suggested that this was caused by their common defect in the TC-NER pathway rather than random fluctuation between the cell lines. To formally prove this in an isogenic model, we repeated the reporter reactivation assay in HeLa cells and the derived monoclonal cell lines, termed “DDB2 ko” and “CSA ko,” where either the GG-NER–specific gene *DDB2* or the TC-NER–specific *CSA* were knocked out by CRISPR-Cas9 interventions (20). The results were highly consistent with findings from the patient-derived cell lines. No differences in the repair capacities were observed between WT and DDB2 ko for all tested *N*^2^-dGuo adducts. In a sharp contrast, disruption of *CSA* resulted in a strong reduction of the EGFP expression both for EtG (from 40.15% ± 3.37% in WT to 9.86% ± 1.72%) and εG (from 71.41% ± 9.02% in WT to 20.29% ± 2.65%) (Fig. 5). An analogous EGFP expression loss was documented for the obligatory TC-NER substrate AAFG in CSA ko, whereas DDB2 ko retained essentially the same repair capacity toward AAFG as WT.

In summary, the results indicate that EtG and εG closely resemble AAFG in their avoidance of GG-NER and significant removal by TC-NER (Figs. 4 and 5). We further note that the rates of reactivation of reporter constructs bearing different modifications (AAFG > εG > EtG) are quantitatively not strictly proportional to transcription blocking capacities of the analyzed adducts in the absence of NER (AAFG > EtG > εG) (Fig. 2). This suggests that structural determinants for transcription blockage and the efficient processing by the downstream TC-NER steps could be different.

## Discussion

**Figure 6:**
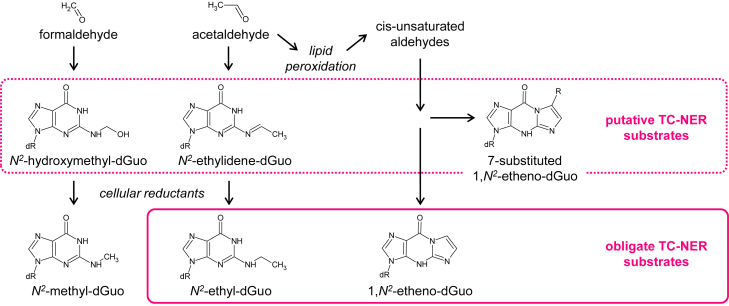
**Obligate TC-NER substrates, *N*^2^-ethyl-dGuoand1,*N*^2^-etheno-dGuo, identified in the present study, and a simplified overview of the structurally related *N*^2^-dGuo adducts generated by formaldehyde, acetaldehyde, and by aldehydic lipid peroxidation products.** dGuo, deoxyguanosine; εG, ethenoguanine; EtG, ethylguanine; TC-NER, transcription-coupled nucleotide excision repair.

Recent research has implicated endogenously arising aldehydes, in particular formaldehyde, in inducing a CS-like phenotype in a TC-NER–deficient mouse model (21). At the molecular level, this finding was recently corroborated by several publications, which reported DNA-protein crosslinks as a cause of formaldehyde-induced toxicity in several independent human cell models (22, 23, 29) and worms (29, 30). Even though CSB and CSA were required for transcription recovery and the relief of the formaldehyde-induced toxicity, the repair mechanism appeared to be different from canonical TC-NER, as it did not require the crucial NER component XPA in either of the reported models. Keeping in mind that formaldehyde and acetaldehyde induce complex spectra of chemically labile DNA adducts (2, 31), it is necessary to consider potential complexity of the involved DNA repair pathways. New results obtained in the present study, using synthetic *N*^2^-alkyl-dGuo adducts, suggest that TC-NER counteracts genotoxicity of DNA damage arising from endogenously generated and dietary aldehydes at the level of several structurally different types of DNA adducts (Fig. 6).

EtG occurs in DNA of alcoholic patients as a natural reduction product of the primary acetaldehyde adduct *N*^2^-ethylidene-dGuo (8), whereas εG is formed, among other mechanisms, by α,β-unsaturated aldehydes emerging from lipid peroxidation under physiological conditions or in response to stressors (4, 32, 33). In particular, increased εG generation has been linked to enhancement of lipid peroxidation as a collateral acetaldehyde toxicity mechanism (2). Our analyses showed that both EtG and εG potently block gene transcription in XP-A cells in a DNA strand–specific manner (Fig. 2), which highlights their capacity to initiate TC-NER. Moreover, the results indicated clear requirements of functional *XPA* (Fig. 3) as well as *CSA* and *CSB* genes (Figs. 4 and 5), but not *XPC* or *DDB2*/*XPE*, for reactivation of the gene expression. Collectively, these results suggest that repair of EtG and εG occurs by canonical TC-NER and essentially by no other mechanism in human cells (Fig. 6).

Instability of primary monoadducts of formaldehyde and acetaldehyde, *N*^2^-hydroxymethyl-dGuo (34, 35) and *N*^2^-ethylidene-dGuo (36, 37), precludes direct assessment of their repair. Nonetheless, extrapolation of results obtained with EtG can be helpful to predict the outcomes of these adducts in transcribed DNA. Based on the close structural similarity to transcription-blocking EtG (14) (Fig. 2), *N*^2^-ethylidene-dGuo (with nearly the same adduct size and an even higher degree of conformational restraint), and *N*^2^-hydroxymethyl-dGuo are reasonably expected to block transcribing RNAPII, which is a prerequisite to TC-NER initiation. Indeed, accumulation of *N*^2^-methyl-dGuo, a reduction product of *N*^2^-hydroxymethyl-dGuo, was reported in organs of *Adh5*^*−/−*^*Csb*^*m/m*^ mice, implying a CSB-dependent TC-NER as a protection mechanism (21). Since *N*^2^-methyl-dGuo does not block transcription to any significant degree (Fig. 2), its recognition by TC-NER is unlikely, which makes us propose *N*^2^-hydroxymethyl-dGuo as a relevant TC-NER substrate within the formaldehyde genotoxicity pathway (Fig. 6).

Although TC-NER-impaired cells are sensitive to formaldehyde (38), we did not detect an increased sensitivity to acetaldehyde in our *CSA* KO model, compared to the isogenic parental cell line (data not shown). A possible explanation is an efficient bypass of acetaldehyde-induced adducts during replication, as inferred from an efficient EtG bypass by human DNA polymerase δ (11). Another explanation could be a high structural complexity of the acetaldehyde adductome, including generation of highly cytotoxic interstrand crosslinks. Based on the available results, we propose that monoadducts arising from primary reactions of aldehydes with DNA are removed by TC-NER, thus preventing immediate transcription-mediated cytotoxic responses. Because of relatively inefficient GG-NER, adducts persist in nontranscribed portions of the genome (or genome-wide in CS patients) and can further react to various types of intermolecular and intramolecular crosslinks, some of which can be converted to cytotoxic damage during DNA replication. Hence, the downstream DNA crosslink repair pathways are of a paramount significance in proliferating cells (39, 40, 41, 42).

Repair mechanisms of εG in mammalian cells were controversially reported in the literature. Human ALKBH2 can repair this lesion in dsDNA in reconstituted biochemical reactions (43). However, ALKBH2 activity toward εG is far lower than toward 1,*N*^*6*^-ethenoadenine, which questioned the relevance of the dealkylation mechanism under physiological conditions in cells, especially taking into consideration that even the relatively good ALKBH2 substrate, 1,*N*^*6*^-ethenoadenine, undergoes repair by AAG-initiated BER rather than by ALKBH2 or ALKBH3 in mouse embryonic fibroblasts (19). Excision by AAG was also initially proposed as a putative mechanism for εG repair (28). However, AAG activity toward εG is almost negligible in comparison to other substrates, including 1,*N*^*6*^-ethenoadenine and hypoxanthine (44). The detected very low activity could be an experimental artefact, since other biochemical studies, corroborated by structural evidence for rejection of guanine modifications in the AAG active site, provided strong evidence against putative relevance of εG as a physiological AAG substrate (45, 46). Finally, NER has been discussed as a putative repair pathway for εG, as its property to potently block transcribing RNAPII was discovered (24). The latter result provides a biochemical background to our finding of persistent and DNA strand–specific transcription blockage by εG in human XP-A cells, which further suggested that repair of this modification is very inefficient or absent in the absence of NER (Figs. 2 and 3). This resembles the behaviour of another exocyclic DNA lesion, 3,*N*^4^-ethenocytosine, which is repaired in mouse cells strictly by TC-NER, as judged by the requirement of *Csa* and *Csb* (19). In the case of εG, the requirement of TC-NER is demonstrated by absent HCR in cell lines derived from CS-A and CS-B patients (Fig. 4) and independently confirmed by reproducing the repair-deficient phenotype by *CSA* knockout in HeLa cells (Fig. 5).

In summary, our discovery of the requirement of *XPA*, *CSA*, and *CSB* for the removal of transcription-blocking EtG and εG (different not only in size, but also in topology and the electron configuration) highlights potential importance of TC-NER as a first-line defence from DNA damage arising from aldehydic compounds from both endogenous and exogenous sources, including the oxidation products of polyunsaturated fatty acids, formaldehyde, and acetaldehyde (Fig. 6). Furthermore, the finding of a strong impact of EtG on gene transcription, despite its relatively small size, merits attention and is in agreement with the RNAPII blockage mechanism reported under cell-free conditions (14). Future systematic investigation should show whether the requirement of TC-NER is a coincidental feature of AAFG, εG, and EtG or is it a hallmark of *N*^2^-alkyl-dGuo adducts as a structural subclass of DNA lesions.

As a limitation of the experimental system employed in the present study and of transiently transfected genetic elements in general, it is necessary to consider that chromatin folding of the plasmid-borne genes is different from the chromosomal DNA. Since NER in the chromosomes requires chromatin rearrangements that still need to be understood, both transcription blockage and repair rates derived from the presented data may not quantitatively reflect the situation in the genome. An invaluable advantage of the plasmid-based system is the possibility of investigation of structurally defined DNA modifications (in our case, physiologically relevant *N*^2^-dGuo adducts or their chemically stable analogs) introduced at a selected specific nucleotide. In this simplified system, DNA strand–specific transcription blockage by synthetic DNA modifications resembling *N*^2^-dGuo adducts induced by aldehydic compounds is clearly observed and the significance of TC-NER–specific genes for the gene expression recovery is shown in human isogenic cell models. It remains to be established whether these damaging agents contribute to the pathogenesis of CS, a severe progeroid and neurodegenerative disease caused by hereditary TC-NER defects.

## Experimental procedures

### Cell lines

The authentic original cell lines were acquired from the specified reliable sources. All cell cultures used in the experiments were routinely tested for *mycoplasma* using Lonza MycoAlert from Biozym Scientific GmbH and were confirmed free from contamination. Immortalized skin fibroblasts derived from patients of different NER complementation groups were XP-A (GM04312) as well as its isogenic cell line obtained by complementation with human XPA cDNA (GM15876), CS-B (GM16095), CS-A (GM16094), and XP-C (GM15983), all purchased from the NIGMS Human Genetic Cell Repository, Coriell Institute for Medical Research. The reference fully repair-proficient fibroblast cell line was MRC-5VA1 (NIA Aging Cell Culture Repository AG10076). Monoclonal HeLa-derived cell lines with disrupted *CSA* and *DDB2* genes, termed “CSA ko” (2C5) and “DDB2 ko” (1D11), were generated and extensively characterized in our laboratory previously (20). Their parental cell line, used as an isogenic reference, was a clone derived from HeLa (German Collection of Microorganisms and Cell Cultures No. ACC 57).

### Synthetic oligonucleotides with *N*^2^-dGuo modifications

Synthetic 18-mer oligonucleotides 5′-CATTGCTTC[G∗]CTAGCACG (where [G∗] is the modification site) contained guanine (G), *N*^2^-MeG, *N*^2^-EtG, 1,*N*^2^-εG, or AAFG. The AAFG oligonucleotide was synthesized by Thomas Carell and co-workers (LMU Munich), as described in detail previously (16). Other strands were purchased from the following manufacturers: G (as an adduct-free reference) was from Eurofins Genomics, MeG from Kaneka Eurogentec, EtG from GeneLink, εG from Baseclick GmbH. All oligonucleotides were HPLC grade. The presence of the specified modifications was confirmed by mass spectrometry, as certified by the suppliers.

### Incorporation of synthetic oligonucleotides into plasmid DNA

Expression vectors pZAJ-5C and pZAJ-5W and their use for site-specific incorporation of synthetic DNA modifications into either the TS or the NTS of the plasmid-borne *EGFP* gene were described previously (26). The two vectors are identical except for the direction of the tandem BsrDI recognition sites and the encompassed 5′-TTCGCTAGCACG sequence in the gene’s 5′ UTR (Figs. 1 and S1). Vectors were nicked by Nb.BsrDI (NEB GmbH) at both sites and were processed to generate 18-nt gaps in the respective DNA strands by depletion of the excised native single-stranded fragment, as described previously (25, 26). In the following step, synthetic 18-mer oligonucleotides containing the specified modifications were annealed to the complementary ssDNA stretch opposite to the gap, 5′-phosphorylated, and ligated to yield fully covalently closed DNA. The proportion of covalently closed plasmid DNA was quantified by analysis of the ethidium bromide–stained agarose gels using the GelDoc XR + Molecular Imager with Image Lab Software (Bio-Rad Laboratories GmbH; https://www.bio-rad.com/de-de/product/image-lab-software).

### Assessment of transcription-blocking capacities of individual adducts in cells and the HCR assay

The methodology for quantitative EGFP expression analyses in transiently transfected cells by flow cytometry was validated over the broad range of gene copy numbers and described previously (47). Cells growing on 6-well plates were cotransfected, using the Effectene transfection reagent (Qiagen), with EGFP reporter constructs (with or without synthetic modifications) mixed with equal amounts of pDsRed-monomer-N1 vector (Clontech) as a gating marker. As in pZAJ-5C and pZAJ-5W, the expression of DsRed-monomer is driven by the human cytomegalovirus immediate early promoter. All plasmids are expressed without integration into chromosomal DNA and do not appreciably replicate in HeLa cells or in the patient-derived cell lines used, as analyzed by differential chromatin fractionation (47), bacterial transformation assays, and retention of Dam methylation for at least 48 h (48).

Cells were fixed at 24 h after transfections and analyzed using a CytoFLEX Flow Cytometer (Beckman Coulter GmbH). Sixty thousand cells were measured per sample and analyzed using the CytExpert software (Beckman Coulter; https://www.beckman.de/flow-cytometry/research-flow-cytometers/cytoflex/software). After exclusion of fragmented and aggregated cells by standard gating procedures, transfected subpopulation was selected based on a preset DsRed fluorescence threshold to plot the EGFP fluorescence distribution and determine its median. The values obtained for constructs carrying each type of *N*^2^-dGuo adduct were used to calculate relative EGFP expression levels with respect to the adduct-free (“G”) control, included in every transfection experiment:

$$relative\;expression^{“}rel\;EGFP^{”}=\frac{median\;EGFP_{adduct}\;}{median\;EGFP_{G}}$$

Modifications were classified as transcription-blocking if (a) the expression in NER-deficient (XP-A) cells was significantly lower than the expression of the adduct-free control (*“rel EGFP” < 1*) and (b) this effect was significantly stronger in the TS than in the NTS. Across different cell lines, recovery of the EGFP expression to a level significantly higher than in the XP-A cell line was defined as “HCR” and interpreted as cells’ repair capacity toward the specific transcription-blocking DNA modification.

### Analysis of the MPG excision activity toward *N*^2^-dGuo adducts

Susceptibility of the investigated *N*^2^-dGuo adducts to excision by the MPG (alias human alkyladenine DNA glycosylase, hAAG) was analyzed under conditions of exhaustive excision of the physiological MPG substrate hypoxanthine. Synthetic deoxyribo-oligonucleotide containing 2′-deoxyinosine (I) was from Kaneka Eurogentec. Covalently closed double-stranded plasmid substrate containing single I residue was produced by a procedure analogous to the incorporation of *N*^2^-dGuo adducts, described above. Oligonucleotides 5′-CATTGCTTCGCT[A∗]GCACG (where [A∗] indicates A or I) were annealed and ligated into the Nb.BsrDI-generated single-stranded gap in the pZAJ-5W plasmid.

Reactions containing 100 ng of the respective covalently closed plasmid constructs, 2 units hAAG (NEB), and 2 units endonuclease IV (NEB) in 15 μl ThermoPol Reaction Buffer (NEB) were incubated for 1 h at 37 °C, followed by heat inactivation for 20 min at 65 °C. Plasmid DNA-containing synthetic apurinic/apyrimidinic site analog tetrahydrofuran was used as a positive control for the endonuclease IV activity, as described previously (18). The reaction products were analyzed by electrophoresis in 0.8% agarose gels containing 0.5 mg/L ethidium bromide, and the incised fraction was quantified with the Image Lab software.

## Data availability

All relevant data are contained within the article. In cases when mean values or representative experiments are reported, data for all individual experiments can be provided by the corresponding author upon request.

## Supporting information

This article contains supporting information.

## Conflict of interest

The authors declare that they have no conflicts of interest with the contents of this article.
