# Supplementary material for: Transcription blocking properties and transcription-coupled repair of N2-alkylguanine adducts as a model for aldehyde-induced DNA damage
Source: J Biol Chem. 2025 Mar 27;301(5):108459. doi: 10.1016/j.jbc.2025.108459 (PMC12051148; doi:10.1016/j.jbc.2025.108459)
Supplement: Supporting information [file mmc1.pdf]

Supporting Information

Title:

Transcription blocking properties and transcription-coupled repair of N^2-alkylguanine adducts as a model for aldehyde-induced DNA damage

Authors:

Leen Sarmini, Nataliya Kitsera, Mohammed Meabed, Andriy Khobta

Material Included:

Figure S1. Page S-1

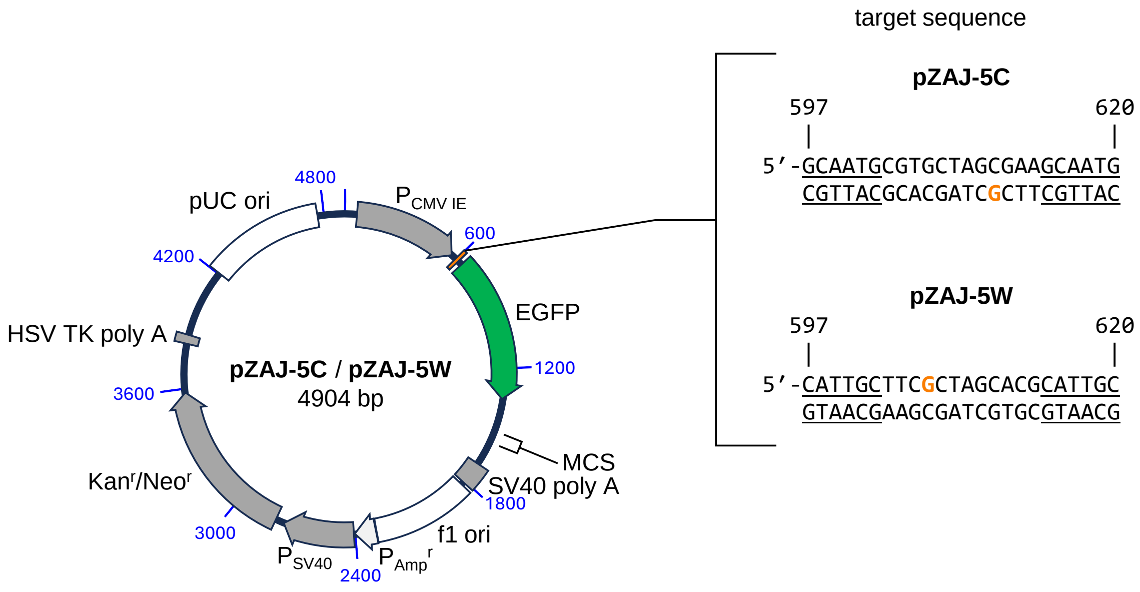

Figure S1. Map of the expression vectors used for site-specific incorporation of N^2-dGuo adducts into the non-coding region of the *EGFP* gene. Both vectors are identical, except for the target sequences displayed in the inset. Modification sites in the transcribed *EGFP* strand (in pZAJ-5C) or in the non-transcribed strand (in pZAJ-5W) are indicated with amber G. Tandem BsrDI sites are underlined. Other functional elements are preserved in both vectors from the original pEGFP-C3 backbone as follows. $P_{CMV\ IE}$: human cytomegalovirus (CMV) immediate early promoter; MCS: pEGFP-C3 multiple cloning site; SV40 poly A: SV40 early mRNA polyadenylation signal; f1 ori: f1 single-strand DNA origin (can package the noncoding strand of EGFP); P_{Amp^r}: bacterial promoter; P_{SV40}: SV40 early promoter; Kan^r/Neo^r: Kanamycin/neomycin resistance gene (neomycin phosphotransferase); HSV TK poly A: Herpes simplex virus (HSV) thymidine kinase (TK) polyadenylation signal; pUC ori: pUC plasmid replication origin.
